# Metabolic Footprint Analysis of Volatile Organic Compounds by Gas Chromatography-Ion Mobility Spectrometry to Discriminate Mandarin Fish (*Siniperca chuatsi*) at Different Fermentation Stages

**DOI:** 10.3389/fbioe.2021.805364

**Published:** 2021-12-31

**Authors:** Yueqi Wang, Yanyan Wu, Yingying Shen, Chunsheng Li, Yongqiang Zhao, Bo Qi, Laihao Li, Yufeng Chen

**Affiliations:** ^1^ Key Laboratory of Aquatic Product Processing, Ministry of Agriculture and Rural Affairs of the People’s Republic of China, National R and D Center for Aquatic Product Processing, South China Sea Fisheries Research Institute, Chinese Academy of Fishery Sciences, Guangzhou, China; ^2^ Co-Innovation Center of Jiangsu Marine Bio-industry Technology, Jiangsu Ocean University, Lianyungang, China; ^3^ Collaborative Innovation Center of Seafood Deep Processing, Dalian Polytechnic University, Dalian, China; ^4^ College of Food Science and Technology, Zhejiang University of Technology, Hangzhou, China

**Keywords:** fermented Mandarin fish, volatile organic compound, gas chromatography-mass spectrometry, electronic nose, metabolic footprint, quality control

## Abstract

Chinese fermented mandarin fish (*Siniperca chuatsi*) have unique aroma characteristics that are appreciated by local consumers. In this study, electronic nose (E-nose) and gas chromatography–ion mobility spectrometry analyses were combined to establish a volatile fingerprint of fermented mandarin fish during fermentation. Clear separation of the data allowed mandarin fish samples at different fermentation stages to be distinguishing using E-nose analysis. Forty-three volatile organic compounds were identified during fermentation. Additionally, partial least squares discrimination analysis was performed to screen for different VOC metabolites in the fermented mandarin fish; the levels of six VOCs changed significantly during fermentation (variable importance in projection >1; *p* < 0.05). Three VOCs, i.e., hexanal-D, nonanal, and limonene were identified as potential biomarkers for fermentation. This study provided a theoretical basis for flavor real-time monitoring and quality control of traditional mandarin fish fermentation.

## Introduction

Spontaneous fermentation is a traditional method for extending the shelf life, improving the flavor, and enhancing the quality of food (Wang et al., 2020). Fermented mandarin fish (*Siniperca chuatsi*) is a traditional Chinese natural fermented food that is typically prepared by adding spices and salt to fresh fish, followed by fermentation in a short-term, low-salt, low-temperature curing process (Shen et al., 2021). Because of its long history and unique regional flavor, fermented mandarin fish is popular among consumers in Asian countries such as Japan, Korea, Thailand, and China. Flavor is an important indicator of the sensory quality of fermented mandarin fish and a key attribute that drives consumer choice (Feng et al., 2021). The production of fermented fish is a time-consuming process, but it is key to the development of the characteristic flavor (Xu et al., 2021). A series of biochemical reactions occur via the action of endogenous enzymes and microorganisms to promote the development of different volatile organic compounds (VOCs) during fermentation, a phenomenon that greatly contributes to flavor development (Flores and Toldrá, 2011). The fermentation of mandarin fish is mainly controlled by modifying the salinity, temperature, and composition. In our previous study, the potential relationship between microorganisms and the production of flavor substances during mandarin fish fermentation was evaluated (Shen et al., 2021). VOCs were generated through protein hydrolysis, lipid degradation and oxidation, Maillard reactions, and Strecker degradation during fermentation. These findings have important implications for exploring the core functional microorganisms involved in this process.

Flavor is an important perceptual characteristic of food. Although direct human sensory analysis is an effective approach for evaluating food flavor, the subjectivity and individual preferences of the subjects limit its utility (Nanda et al., 2021). Further development of molecular sensory analysis technologies may overcome these limitations. The molecular sensory techniques used to analyze food flavor include gas chromatography-mass spectrometry (GC-MS), gas chromatography-olfactory-mass spectrometry, gas chromatography–ion mobility spectrometry (GC-IMS), and electronic nose (E-nose) analysis (Pérez-Jiménez et al., 2021). GC-MS is currently the most versatile method for analyzing volatile compounds in food science, but it requires complex sample preparation procedures and the generation of vacuum, which prolongs the analysis time; therefore, this method is not suitable for rapidly characterizing VOCs in food products (Wang et al., 2020). In contrast, ion mobility spectroscopy is a trace chemical analytical technique that separates ionized compounds in a neutral gas phase at atmospheric pressure, thereby enabling the rapid identification of isobaric and isomeric compounds. It detects trace VOCs within different matrices with high sensitivity (Armenta et al., 2011). GC-IMS is a newly developed and powerful technique for studying the flavor profiles of fermented products, including dry-cured pork (Li et al., 2021), olive oil (Garrido-Delgado et al., 2015), and *Tricholoma matsutake* (Li et al., 2019). Recently, a method using an E-nose and headspace-GC-IMS was established to detect VOCs in aquatic products and analyze the effects of four heat-treatment methods on the aroma characteristics of tilapia muscles treated with acidity regulators (Chen et al., 2021).

Previous studies have shown that the volatile metabolic profiles of fermented mandarin fish differed with fermentation time (Shen et al., 2021). However, little is known about the specific volatile metabolites produced. Studying these metabolites would provide valuable insight into the time-specific profiles of VOCs. In summary, the similarities and differences in the VOCs of fermented mandarin fish remain unclear, and no validated models are available for accurately predicting the trends in the natural fermentation processes. GC-IMS combined with E-nose technology enables intuitive comparisons across samples based on establishing a fingerprint of VOCs during mandarin fish fermentation. Therefore, in this study, we estimated the differences in VOCs during mandarin fish fermentation using E-nose analysis and GC-IMS to assess the abilities of these methods to predict the degree of natural fermentation. Subsequently, the calculated variable importance in projection (VIP) scores obtained from partial least squares discriminant analysis (PLS-DA) were used to identify potential biomarker VOCs during fermentation. We performed a detailed evaluation of mandarin fish fermentation to facilitate the development of improved strategies for targeted flavor regulation and quality monitoring in Chinese fermented mandarin fish.

## Material and Methods

### Sampling of Fermented Mandarin Fish Products

Traditional fermented mandarin fish were collected from a fish processing plant in Anhui Province, China, as described previously (Shen et al., 2021). The raw mandarin fish were 28 ± 3.0 cm in length and 500 ± 50.0 g in weight. Whole fish were gutted, washed, and then carefully stacked layer by layer in a fermentation barrel. Next, approximately 5% salt and 0.02% spices were added and mixed with the fish. The fish was fermented at 20 ± 5°C and 45 ± 15% relative humidity under natural environmental conditions. To eliminate the influence of external bacteria during sampling, the entire sampling process was performed on an ultraclean sterile bench. The samples were collected at 0 days (0D), 4 days (4D), 8 days (8D) and 12 days (12D) for further measurement. Five fermented mandarin fishes were taken from each time point for a parallel experimental group. The muscle from the dorsal side of the fish was sampled and then frozen at −18°C for further analyses.

### E-Nose Analysis

The overall odor profiles of the samples were identified using a Fox 4,000 gas-sensor E-nose (Alpha MOS Co., Toulouse, France) equipped with 18 metal-oxide sensors. The sensor array system was used to monitor the VOCs in the samples; the function of each sensor is listed in Supplementary Table S1. E-nose determination of the fermented mandarin fish was performed as described by Pei et al., with slight modifications (Pei et al., 2016). Samples (1.0 g) were placed in a 15-ml headspace bottle and balanced in a metal bath for 10 min at 75°C. The samples were injected into the Fox system using an autosampler at 60°C. The acquisition time was 120 s with a flow rate of 150 ml/min and an injection volume of 1 ml. After the measurement of each sample was completed, the device was purged with clean air for 10 min to prevent sample odor residue, and then the next sample was automatically analyzed. Four replicate measurements were made for each sample.

### GC-IMS Analysis

The VOCs in traditional fermented mandarin fish were identified using a FlavourSpec GC-IMS (G.A.S., Dortmund, Germany) equipped with a syringe and an autosampler unit for headspace analysis. Briefly, 2 g of homogenized sample was transferred into a 20-ml headspace vial, and the vial was sealed using a magnetic cap with a silicone septum (Chen et al., 2021). Next, 500 µl of the headspace volume was injected automatically into the heating injector with a heated syringe at 85°C, and the incubation rotation speed was maintained at 500 rpm. Nitrogen (99.99% pure) was used as a carrier gas to transport the sample into a MXT-5 capillary column (15 m × 0.53 mm ID) (Restek Corp., Bellefonte, PA, United States ) for separation at a flow rate of 150 ml/min. The column temperature was maintained at 60°C, and the IMS temperature was 45°C. The carrier gas flow rate was set to 2 ml/min for 2 min and subsequently increased to 100 ml/min for 18 min (Song et al., 2021). The VOCs were identified by comparing their cation retention index and drift time with information in the FlavourSpec GC-IMS library and NIST 2014 databases (G.A.S, Dortmund, Germany).

### Statistical Analysis

Data acquired using GC-IMS and E-nose were visualized and analyzed using the commercial software LAV 2.2.1 (G.A.S.) and Alphasoft V14 (Alpha MOS), respectively. Before exploratory or categorical analysis, various data preprocessing methods (i.e., mean centering, autoscaling, Pareto scaling, and transformation) were tested individually and in combination to highlight relevant changes contained in the data. Heat maps were constructed using TBtools (Toolbox for Biologists, version 1.082, China). PLS-DA was performed using MetaboAnalyst version 5.0 (Pang et al., 2021). All experiments were performed in triplicate, except for the E-nose test, which was repeated four times.

## Results and Discussion

### Flavor Profile Dynamics During Mandarin Fish Fermentation

**FIGURE 1:**
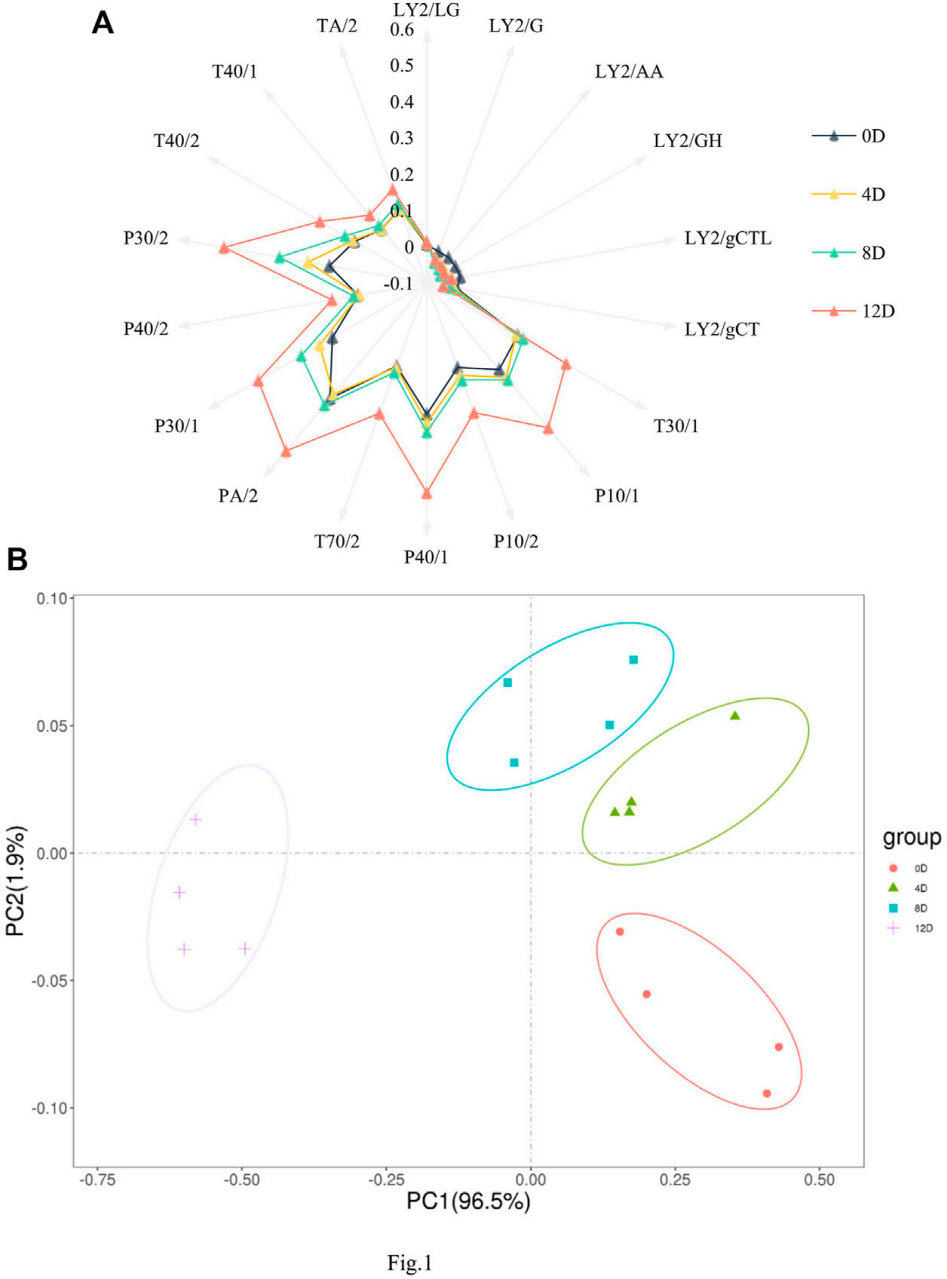
Radar charts. **(A)** and principal component analysis plot. **(B)** of the flavor profiles of fermented mandarin fish. Values are obtained from four biological replicates.

The E-nose was used to detect and determine flavor profile dynamics during mandarin fish fermentation. Sensors in the E-nose can simulate the sense of smell of organisms (Di Rosa et al., 2017) and can thus distinguish various flavors during fermentation. A radiation map was established by extracting the response strength of each sensor (Figure 1A). Specifically, most sensor responses showed a gradually increasing trend, with sensors PA/2, P40/1, P30/2, P30/1, P10/1, and T30/1 showing significantly higher values at 12 days than at any other fermentation stage, indicating that the levels of hydrocarbons, alcohols, nitrogenous compounds, and other compounds in the samples changed during fermentation.

The spatial distribution areas in the principal component analysis (PCA) plot reflected the differences between the samples. All samples were distributed in different regions, with no overlap. As shown in Figure 1B, the first and second principal components explained 98.4% of the total variance, and the E-nose combined with PCA effectively distinguished the mandarin fish samples at different fermentation stages. In agreement with our results, Yao et al. (2015) clearly and effectively distinguished flavor differences in traditional fermented vegetable samples subjected to different salting times using the E-nose technique. Drake et al. (2003) showed that E-nose combined with descriptive sensory analysis can be used to screen and differentiate aged cheddar cheese from various sources after different fermentation times. Based on the different odor characteristics of the fermented fish, we successfully distinguished mandarin fish samples at different fermentation stages. In the PCA plot, the 0 day sample was distant from the other samples, mainly because of the flavor conferred to the product by fermentation; additionally, the overall odor was similar at 4, 8, and 12 days, revealing significant differences between the fermented and raw samples.

### Dynamic Changes in Flavor Components During Mandarin Fish Fermentation

**FIGURE 2:**
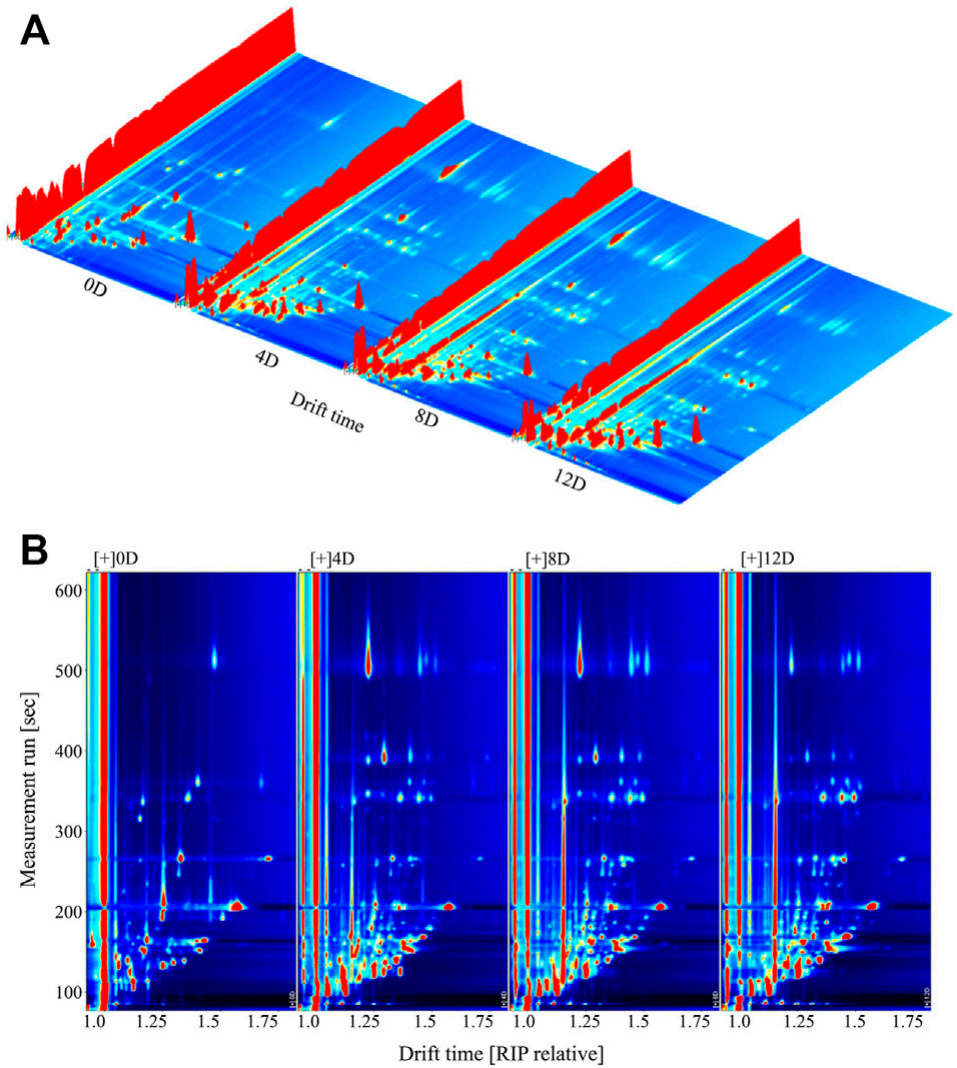
Three-dimensional. **(A)** and two-dimensional. **(B)** topographic plots of volatile compounds in fermented mandarin fish. Values are obtained from three biological replicates.

The VOC content of mandarin fish during fermentation was detected using GC-IMS. The data were visually represented in a three-dimensional (3D) topographic plot, where the *x*-, *y*-, and *z*-axes represented the ion migration time for identification, the retention time of the gas chromatograph, and the peak height of quantification, respectively (Arroyo-Manzanare et al., 2018). As shown in Figure 2A, the VOC composition of the samples changed significantly during fermentation. GC-IMS separation is based on the strength of the forces acting on the VOC. Eluted components with different retention times are ionized in gaseous form by the ion source; the migration rates differ because of differences in the mass, collision cross section, charge, and periodic ion pulses of the ion groups, resulting in secondary effective separation (Wang S. et al., 2020). The peak signal distribution was significantly lower in fresh mandarin fish (0 days) than in fish at other fermentation stages, revealing that all the fermented samples released more flavor components, with slightly different signal intensities. The samples at day 4 and 8 showed similar results, and the signal intensity of the same flavor compounds showed different development trends after 12 days of fermentation.

The 3D topography was rather coarse and unsuitable for data observation. Therefore, we normalized the ion migration time and position of the reactive ion peak to obtain an aerial view of the topographic plots (Figure 2B). The entire topographic plot presented all the flavor compounds in the samples, and each point represented the VOCs identified from the fermented mandarin fish. A white background indicated that the compound was consistent with the reference; red and blue backgrounds indicated compounds with higher and lower signal intensities than the reference, respectively (Li et al., 2019). Most signals appeared at a retention time of 100–500 s (drift time = 1.0–1.5 s). Numerous red spots were observed on the plots of the mandarin fish samples fermented for 4, 8, and 12 days, indicating that the signals of most of the VOCs were much higher after fermentation than those of the fresh sample. Moreover, several blue spots were observed on the plots of the samples that underwent fermentation for 4–12 days, revealing that some flavor compounds caused the disappearance or reduction of signals.

### Identification of Flavor Compounds During Mandarin Fish Fermentation

**FIGURE 3:**
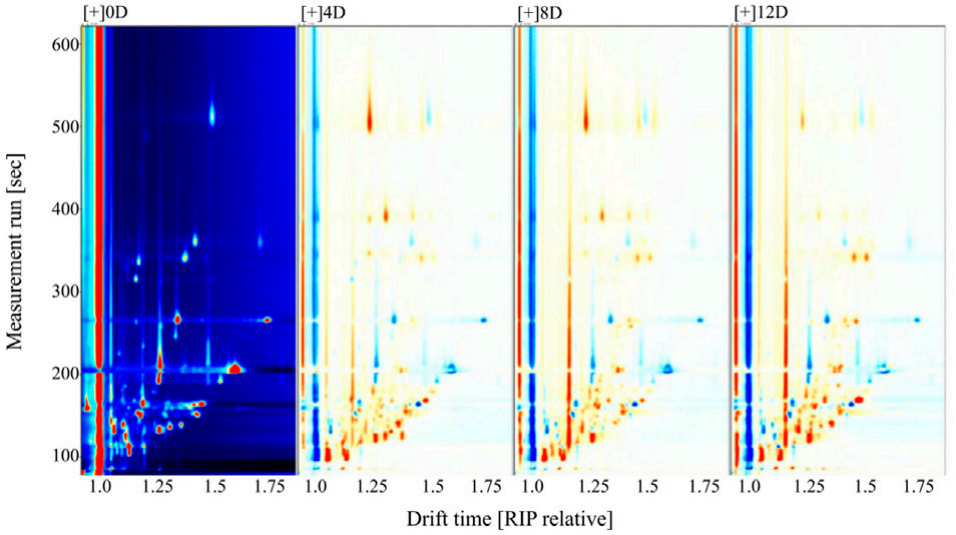
Comparative difference spectra of volatile compounds in fermented mandarin fish. Values are obtained from three biological replicates.

**FIGURE 4:**
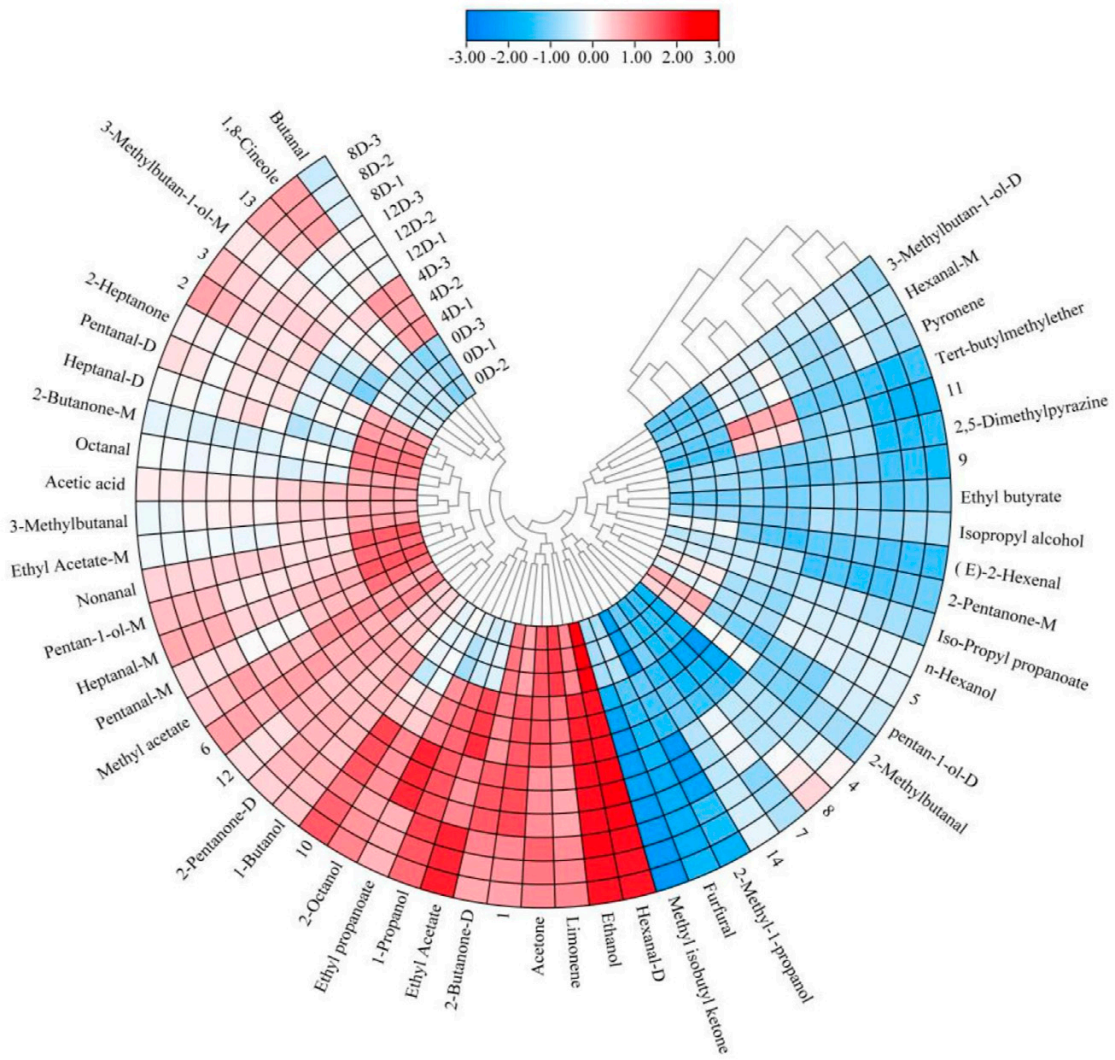
Heat map and hierarchal clustering of the volatile profiles of fermented mandarin fish samples. Values are obtained from three biological replicates.

Fifty-seven VOCs were identified from the fermented mandarin fish, among which 43 were included in the databases (Supplementary Table S2). To improve our understanding of the variations in flavor compounds in the samples during fermentation, overlapping differentiation spectra were analyzed (Figure 3). We used the 0-days sample as a reference to compare the differences in VOC between the raw material and fermented samples. A red color indicated that the concentration of a substance in the sample was higher than that in the reference sample, whereas a blue color indicated that the concentration was lower than that in the reference sample. Heat map clustering analysis was performed to further elucidate the VOC composition of mandarin fish at different fermentation stages. The samples divided into two categories as the squared Euclidean distance increased, with the four fermentation stages showing different characteristic VOCs (Figure 4). Owing to the high proton affinity of certain compounds, the ions may form dimers or trimers upon transfer to the drift tube (Zhang et al., 2022). This suggests that the trend in monomer variation differs based on the dimerization of the same molecule, even under the same fermentation conditions.

**FIGURE 5:**
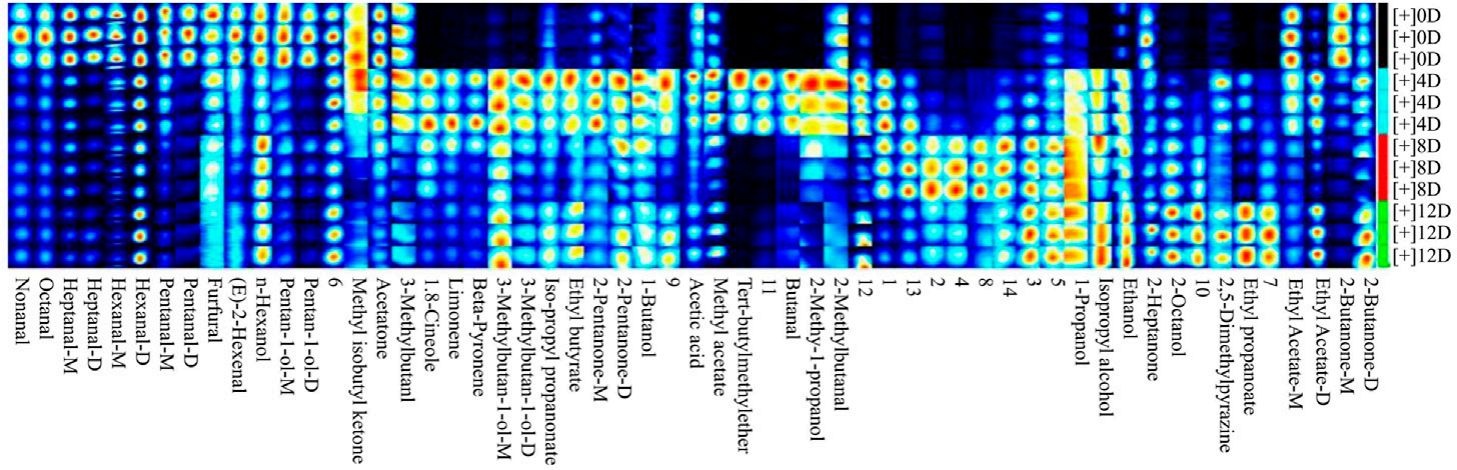
Gallery plot fingerprint profiles of volatile compounds in fermented mandarin fish. Values are obtained from three biological replicates.

The fingerprint profile (Figure 5) showed that 47 VOCs, including 13 aldehydes, 12 alcohols, seven ketones, seven esters, two hydrocarbons, one pyrazine, and one ether were produced during the different fermentation stages. Aldehydes were the most diverse compounds detected and were mainly produced via the hydrolysis of proteins and the oxidation of unsaturated fatty acids during fermentation (Wang et al., 2020). Straight-chain aldehydes were the most abundant aldehydes in the fermented mandarin fish and mainly included nonanal, heptanal (monomer and dimer), hexanal (monomer and dimer), pentanal (monomer and dimer), and octanal (Supplementary Table S2). Hexanal is among the most abundant aldehydes and is formed by the oxidation of *n*-6 fatty acids, such as linoleic acid and arachidonic acid (Hu et al., 2021). The production of nonanal, heptanal, and pentanal may also be related to lipid oxidation (de Lima Alves et al., 2020). Additionally, the content of these aldehydes decreased after fermentation, which was consistent with the results of Yang et al. (2020). They proposed that this was mainly caused by protein hydrolysis, which accelerated the formation and release of flavor substances to improve the overall flavor and aroma of the product. However, hexanal, heptanal, and nonanal still had an important impact on the flavor of fermented mandarin fish because of their low thresholds and grass-like, molasses, and nut-like aromas. Two methyl branched aldehydes (2-methylbutanal and 3-methylbutanal) were detected in the fermented mandarin fish samples; these were derived from the Streker degradation reaction of amino acids (Afzal et al., 2012). Similar results were obtained in an analysis of the flavor components of shrimp paste; the compounds 2-methylbutanal and 3-methylbutanal imparted chocolate, almond, and coffee flavors to the product (Zhu et al., 2019).

Alcohols are the key aroma-imparting components in traditionally fermented fish (Wang et al., 2021); ethanol, 1-propanol, and 2-octanol are the three dominant alcohols present. Ethanol is mainly derived from glucose metabolism through alcohol fermentation or via the pentose-phosphate route (de Lima Alves et al., 2020). The signal intensities of these compounds were enhanced by fermentation, and those of 3-methylbutan-1-ol (monomer and dimer), 2-methyl-1-propanol, 1-butanol, 1,8-cineole, and isopropyl alcohol were also increased. 2-Methyl-1-propanol and 1-butanol can be produced via the metabolism of valine, which is an important VOC in fermented black glutinous rice wine (Zhao et al., 2020). Non-branched alcohols, such as 1-butanol and 1-propanol, can contribute to a grassy or woody odor. 3-Methylbutan-1-ol is produced by leucine deamination and decarboxylation reactions associated with microbial activity (Englezos et al., 2018).

The ketone production pathway is similar to that of aldehydes and is mainly based on amino acid degradation and unsaturated fatty acid oxidation. During the formation of aldehydes and ketones, α-keto acids act as key nodal compounds in the amino acid metabolic pathway to produce correspondingly different aldehydes and ketones by the action of different branched-chain amino acid transaminases (Wang et al., 2020). The signal intensities of most ketones in the fermented mandarin fish showed decreasing trends, including those of 2-heptanone (monomer and dimer), methyl isobutyl ketone, and 2-butanone (monomer). The signal intensities of acetone and 2-pentanone (monomer) were the highest on day 4, whereas they were weaker than those of fresh samples after 8 days. Glycogen in the muscle reacts with amino acids, peptides, and proteins to form aliphatic compounds with fewer than six carbon atoms, such as acetone (Shi et al., 2019). For example, alcohols can be converted via intermediate reactions between ketones and aldehydes, resulting in decreases in the levels of these compounds after fermentation. Additionally, the levels of 2-pentanone (dimer) and 2-butanone (dimer) changed dynamically throughout the fermentation process, but its signal at the end of fermentation was higher than that of fresh samples (Supplementary Table S2). Compared to the results of a previous study that aimed to identify VOCs in fermented mandarin fish using GC-MS (Li et al., 2013), more ketones were identified in this study, indicating that GC-IMS is more sensitive and able to identify more trace VOCs than GC-MS.

Ester VOCs in fermented aquatic products are predominantly fatty acid ethyl esters, which are formed mainly by microbial enzymatic esterification reactions (Khan et al., 2015; Wang Y et al., 2020). Ester VOCs, which have a low detection threshold, are important compounds that increase the fruity and sweet aroma of traditional fermented mandarin fish. The signal intensities of most esters, including ethyl butyrate, isopropyl propanoate, ethyl propanoate, ethyl acetate (dimer), and methyl acetate, were stronger in fermented samples than in fresh samples. Ethyl esters, including ethyl acetate (monomer and dimer), ethyl butyrate, and ethyl propanoate were also detected in the fermented mandarin fish. Ethanol produced during fermentation can be used as a substrate for esterification, which may be associated with the presence of microbial enzymes. Acetic acid was also identified in the fermented samples; this compound is known to greatly affect flavor. Additionally, limonene was identified, which was consistent with our previous findings that limonene was mainly introduced through the addition of flavoring during fermentation, thereby conferring fruity and floral aromas to the product (Wang et al., 2021).

### Screening for Potential Biomarker VOCs for Fermented Mandarin Fish

**FIGURE 6:**
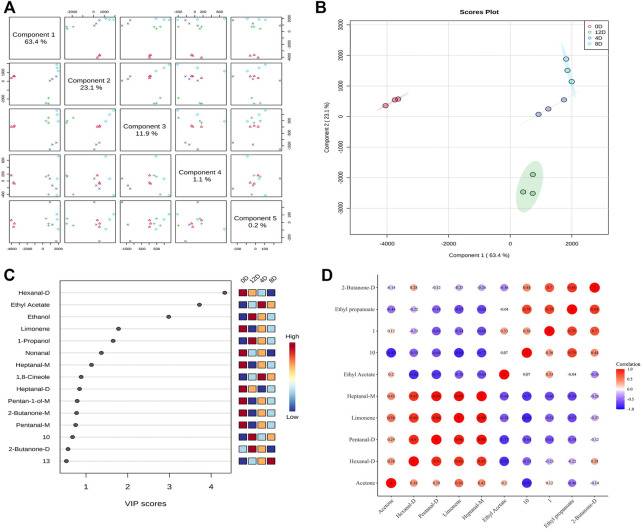
Overview of score plots. **(A)** and two-dimensional score plots. **(B)** of fermented mandarin fish samples based on partial minimum discriminant analysis. Important features of mandarin fish during fermentation identified by partial minimum discriminant analysis. **(C)** Colored boxes on the right indicate the relative concentrations of the corresponding metabolites in each evaluated group. Correlation map of volatile metabolites (VIP >1) subjected to fermentation. **(D)** Values are obtained from three biological replicates.

A PLS-DA model was established to screen and identify potential biomarker VOCs for fermented mandarin fish. PLS-DA is a supervised multivariate data analysis method that reflects the relationship between metabolite expression and sample categories for sample category prediction (Biancolillo et al., 2021). The *R* ^2^ and Q^2^ values of the PLS-DA model were 0.80 and 0.63, respectively, indicating the excellent applicability and predictability of this model and that the model was reliable without overfitting of data. The score chart (Figures 6A,B) showed that the fermented mandarin fish samples had regional distribution characteristics and were well-distributed in space. These data suggest that microbial fermentation significantly impacted the metabolite composition of mandarin fish during fermentation. The VIP scores obtained from the PLS-DA model can be used to measure the strength of impact and explanatory power of various volatile metabolites based on the classification and differentiation of each sample (Giannetti et al., 2019). Therefore, we combined the VIP scores and correlation matrix to explore the relationships of VOCs during mandarin fish fermentation. Substances with VIP scores >1 were selected for further model construction and the screening of potential VOC biomarkers. Eight discriminatory volatiles were identified: hexanal-D, ethyl propanoate, limonene, ethyl acetate, heptanal-M, pentanal-D, acetone, and 2-butanone-D (Figure 6C). Hexanal, heptanal, and pentanal were mainly derived from lipid oxidation and degradation. These compounds exhibited grassy and fatty aromas and strongly influenced the overall flavor because of their low detection threshold values, similar to ethyl propanoate and ethyl acetate, which increased the fruity and sweet aromas of the fermented mandarin fish. Amino acids can also be degraded by microorganisms via Strecker degradation to produce short-chain aldehydes (Liu et al., 2021). Ester compounds are mainly formed by microbial enzymatic reactions, and volatile esters can be produced by esterification of the corresponding alcohols and fatty acids (Gurav and Bokade, 2010).

To assess the interdependence of the various VOCs, a correlation matrix was used to determine correlations between the levels of volatile flavors in samples collected at different fermentation stages; the color depth correlated with the absolute value of the correlation coefficient, and the point size correlated with the significance of the correlation (Figure 6D). Four substances, hexanal-D, nonanal, limonene, and heptanal-M, were positively correlated with each other. Moreover, 2-butanone-D was positively correlated with ethyl propionate. Notably, ethyl propanoate was negatively correlated with most aldehydes (hexanal-D, pentanal-D, and heptanal-M). We previously assessed the VOCs in Chinese fermented mandarin fish products using headspace solid-phase microextraction GC-MS. We found that 1-octen-3-ol, limonene, nonanal, and hexanal contributed significantly to flavor formation in fermented mandarin fish (Wang et al., 2021). These compounds impart fruity, fatty, floral, and fishy flavors to the fish. Combined with our previous results that depicted changing trends in the VOC content of Chinese fermented mandarin fish, the present results identified hexanal-D, nonanal, and limonene as potential biomarker VOCs for effectively predicting the degree of fermentation and flavor quality of mandarin fish.

Furthermore, our study indicated that GC-IMS fingerprinting was superior to E-nose analysis, for monitoring the dynamic changes in fermented mandarin fish flavor. This is attributed to the high separation capacity of GC and the high molecular specificity synergy of IMS. The low detection threshold and odor visualization advantages of GC-IMS at the parts per billion in volume (ppbv) level would also facilitate its successful practical application in the real-time quality monitoring of food products (Wang et al., 2020). The VOCs that showed similar trends during mandarin fish fermentation were mainly alcohols, esters, and aldehydes. Further studies are required to develop electrochemical sensors with high molecular specificity for biomarker VOCs to provide a portable, rapid, and accurate tool for specialized detection. Additionally, a broader range of fermentation environments, species, and fermented fish production methods should be considered, as these may affect the VOC profiles of fermented foods. This research provides theoretical support for targeted flavor regulation during the processing of fermented fish.

## Conclusion

In this study, GC-IMS combined with E-nose analysis was used to comprehensively determine the flavor development of mandarin fish products during fermentation. Clear separation of the data allowed the discrimination of mandarin fish samples at different fermentation stages based on the results of E-nose analysis. A total of 43 VOCs were identified in the fermented mandarin fish samples. Moreover, a PLS-DA model was established to screen for different VOC metabolites. Hexanal-D, nonanal, and limonene were identified as potential biomarker VOCs to effectively predict the flavor quality of Chinese fermented mandarin fish. This research provides theoretical support for the targeted flavor regulation and product quality monitoring of fermented mandarin fish, which may lead to improvements in the traditional fermented fish industry.

## Data Availability

The original contributions presented in the study are included in the article/Supplementary Material, further inquiries can be directed to the corresponding authors.

## Abbreviations

E-nose, electronic nose; GC-IMS, gas chromatography–ion mobility spectrometry; GC-MS, chromatography-mass spectrometry; PCA, principal component analysis; VIP, variable importance in projection; VOC, volatile organic compounds.
