# Supplementary material for: Metabolic Footprint Analysis of Volatile Organic Compounds by Gas Chromatography-Ion Mobility Spectrometry to Discriminate Mandarin Fish (Siniperca chuatsi) at Different Fermentation Stages
Source: Front Bioeng Biotechnol. 2021 Dec 31;9:805364. doi: 10.3389/fbioe.2021.805364 (PMC8758571; doi:10.3389/fbioe.2021.805364)
Supplement: Supplementary file 1 [file Table1.docx]

Table S1 Sensors and sensitive compounds of electronic nose

| Sensors | Sensitive compounds |
| --- | --- |
| LY2/LG | Oxynitride, sulfide, chloride, fluorine |
| LY2/G | Carbon oxide, amines, ammonia |
| LY2/AA | Ammonia, ethanol, acetone |
| LY2/GH | Amines, ammonia |
| LY2/gCTL | Hydrogen sulfide |
| LY/gCT | Propane, butane |
| T30/1 | Chloride |
| P10/1 | Hydrocarbon, ammonia, chlorine |
| P10/2 | Methane, ethane |
| P40/1 | Chlorine, fluorine |
| T70/1 | Toluene, xylene, carbon oxide |
| PA/2 | Amines, ammonium hydroxide, ethanol |
| P30/1 | Hydrocarbon, ammonia, ethanol |
| P40/2 | Hydrogen sulfide, chlorine, fluorine |
| P30/2 | Ketone, hydrogen sulfide |
| T40/2 | Chlorine, fluorine |
| T40/1 | Fluorine |
| TA/2 | Ethanol |
