# Supplementary material for: Metabolic Footprint Analysis of Volatile Organic Compounds by Gas Chromatography-Ion Mobility Spectrometry to Discriminate Mandarin Fish (Siniperca chuatsi) at Different Fermentation Stages
Source: Front Bioeng Biotechnol. 2021 Dec 31;9:805364. doi: 10.3389/fbioe.2021.805364 (PMC8758571; doi:10.3389/fbioe.2021.805364)
Supplement: Supplementary file 2 [file Table2.docx]

Table S2 Volatile compounds of fermented mandarin fish

| Count | Compound | RI | Rt [sec] | Dt [a.u.] | Comment |
| --- | --- | --- | --- | --- | --- |
| 3 | Nonanal | 1113.2 | 513.753 | 1.47621 |  |
| 4 | 1.8-Cineole | 1029 | 392.715 | 1.29527 |  |
| 5 | Octanal | 1007.5 | 361.731 | 1.40472 |  |
| 6 | 2-Octanol | 992.2 | 342.254 | 1.44331 |  |
| 8 | Heptanal-D | 902 | 265.333 | 1.70893 | Dimer |
| 9 | 2-Heptanone | 895.2 | 259.49 | 1.26279 |  |
| 11 | n-Hexanol | 874.1 | 247.248 | 1.32342 |  |
| 13 | 2,5-Dimethylpyrazine | 894.5 | 258.934 | 1.11637 |  |
| 15 | Heptanal-M | 903 | 266.145 | 1.33021 | Monomer |
| 16 | Limonene | 1026 | 388.29 | 1.22719 |  |
| 17 | Beta-Pyronene | 996.9 | 346.574 | 1.2247 |  |
| 18 | Hexanal-M | 801.9 | 208.549 | 1.25787 | Monomer |
| 19 | Hexanal-D | 796.5 | 205.638 | 1.57212 | Dimer |
| 20 | (E)-2-Hexenal | 850.8 | 234.746 | 1.18506 |  |
| 21 | Furfural | 833.4 | 225.431 | 1.08669 |  |
| 22 | Pentan-1-ol-M | 773.2 | 194.578 | 1.2502 | Monomer |
| 23 | Pentan-1-ol-D | 769.6 | 193.122 | 1.50825 | Dimer |
| 24 | Pentanal-M | 700.2 | 164.982 | 1.18442 | Monomer |
| 25 | Pentanal-D | 698.7 | 164.393 | 1.4295 | Dimer |
| 26 | Methyl isobutyl ketone | 736.5 | 179.714 | 1.18056 |  |
| 27 | 3-Methylbutan-1-ol-M | 737.8 | 180.238 | 1.24059 | Monomer |
| 28 | 3-Methylbutan-1-ol-D | 735.7 | 179.367 | 1.49344 | Dimer |
| 29 | iso-Propyl propanoate | 754.8 | 187.136 | 1.18245 |  |
| 30 | Ethyl butyrate | 803.3 | 209.292 | 1.20498 |  |
| 31 | Ethyl propanoate | 710.1 | 169.008 | 1.46414 |  |
| 32 | 2-Pentanone-M | 690.4 | 161.028 | 1.12388 | Monomer |
| 35 | 2-Pentanone-D | 693.7 | 162.373 | 1.38261 | Dimer |
| 36 | 1-Butanol | 669.1 | 155.03 | 1.38369 |  |
| 38 | 3-Methylbutanal | 655.4 | 151.336 | 1.41587 |  |
| 39 | Ethyl Acetate-D | 614.2 | 140.235 | 1.34838 | Dimer |
| 40 | Acetic acid | 636.5 | 146.245 | 1.04741 |  |
| 41 | Ethyl Acetate-M | 620.2 | 141.853 | 1.10398 | Monomer |
| 42 | 2-Butanone-D | 590.2 | 133.763 | 1.25411 | Dimer |
| 43 | 1-Propanol | 550 | 122.899 | 1.2548 |  |
| 45 | Methyl acetate | 523.8 | 115.849 | 1.19056 |  |
| 46 | Isopropyl alcohol | 518.7 | 114.462 | 1.0935 |  |
| 47 | Acetone | 520.4 | 114.924 | 1.12423 |  |
| 48 | Ethanol | 476.3 | 103.02 | 1.13121 |  |
| 49 | 2-Butanone-M | 592 | 134.225 | 1.06347 | Monomer |
| 50 | Tert-butylmethylether | 560.7 | 125.788 | 1.36304 |  |
| 52 | Butanal | 559.8 | 125.557 | 1.29251 |  |
| 53 | 2-Methyl-1-propanol | 635.2 | 145.898 | 1.36444 |  |
| 57 | 2-Methylbutanal | 665.9 | 154.168 | 1.40289 |  |
